# By-Products of the Babassu Agribusiness for Ruminant Diets

**DOI:** 10.1155/2024/5363940

**Published:** 2024-08-05

**Authors:** Ygor Nascimento Portela, Danrley Martins Bandeira, Daniele de Jesus Ferreira, Henrique Nunes Parente, Michelle de Oliveira Maia Parente, Ivo Alexandre Leme da Cunha, Glayciane Costa Gois, Fleming Sena Campos, Francisca Claudia da Silva de Sousa, Luana Milena Pinheiro Rodrigues, Jessica Maria de Sousa Oliveira, Anderson de Moura Zanine

**Affiliations:** ^1^ Universidade Federal do Maranhão Post-Graduate Program in Animal Science, Chapadinha, MA 65500-000, Brazil; ^2^ Universidade Federal do Piauí Post-Graduate Program in Tropical Animal Science, Teresina, PI 64049-550, Brazil; ^3^ Universidade Federal da Paraíba Post-Graduate Program in Animal Science, Areia, PB 58397-000, Brazil

## Abstract

The use of babassu agro-industrial residues in animal feed, in addition to being an economic option of great importance in reducing the environmental impact in regions of the Brazilian Cerrado, provides the production of good quality foods of animal origin due to its nutritional characteristics. However, information related to the nutritional components of babassu by-industrial residues has not yet been sufficiently explored. The aim of this study was to evaluate the nutritional potential of some by-products from the babassu production chain through chemical composition and in situ degradability analyses. The experiment was conducted in a completely randomized experimental design, with 4 by-products from babassu processing (cake, greasy, fine flour, and 95 *µ*m flour) and 5 replications. The by-products differ in terms of chemical composition, except for the hemicellulose content. For the degradability of fraction “a” of dry matter, it presented a higher percentage for 95 *µ*m flour. Fine flour and 95 *µ*m flour presented the highest fractions “b” and “c,” potential, and effective degradability of dry matter. For the degradation of crude protein, the highest percentages of potential and effective degradability were observed for greasy and 95 *µ*m flour. The highest standardized potentially degradable fraction and the highest passage rate were obtained by 95 *µ*m flour, which also showed greater degradation for dry matter, crude protein, and neutral detergent fiber. Among the by-products studied, the babassu cake has superior chemical composition; however, the 95 *µ*m flour presented nutritional value and satisfactory rumen degradation to be used as an additive or in partial replacement of traditional concentrates.

## 1. Introduction

The continuous search for alternative feeds that can replace the traditionally used feeds in formulating diets for ruminants, aiming to reduce costs while maintaining or even enhancing animal performance, is a major concern for animal nutritionists [1, 2]. Therefore, various agribusiness by-products have shown potential for use in ruminant feeding due to their nutritional components such as carbohydrates, proteins, minerals, fibers, vitamins, antioxidants, and bioactive compounds, which are essential for animal development, as well as their ability to improve the quality of final products (meat and milk) [3–6].

Technological innovations aimed at enhancing the efficiency of animal production systems are necessary to sustainably address global food security demands [7]. As reported by Goenag et al. [8], for every ton of fruits and vegetables produced within the food chain worldwide, approximately 10–60% of losses occur during processing, resulting in 6 million tons of solid waste and by-products annually. Therefore, the introduction of these by-products into ruminant diets represents a significant opportunity for reducing feeding costs, considering the seasonality of forage production [9, 10], and consequently achieving greater profitability for the production chain. Additionally, it provides a viable solution for the disposal of by-products, as the vast majority of them lack established applications, are considered surplus in the production chain, and are likely contributors to environmental issues [4, 8].

Babassu palm (*Attalea speciosa*) is cultivated throughout the Americas, Africa, Asia, and the Middle East [11]. In Brazil, babassu is mainly found among the states in the North-Northeast regions, with the highest occurrence in the babassu palm forests, a transitional region between the Amazon rainforest and the semiarid region of Brazil [12]. In the state of Maranhão, approximately 10 million hectares of babassu are found, accounting for about 94% of the national production [13].

Babassu is considered the world's largest source of wild seed oil, accounting for approximately 60 to 72% of the weight of the almond. However, industrial extraction of babassu oil generates 337 kg of by-products per ton of almonds used [12]. The generated by-products (cake, bran, and flour from babassu mesocarp) have been increasingly used in animal nutrition as alternative feed or in replacement of other traditional feeds [11, 12, 14–17].

Therefore, determining the chemical and bromatological composition, as well as the kinetic parameters of ruminal degradation of these by-products, is of utmost importance in the nutritional context, given that they have not yet been fully elucidated. This information serves as the starting point for formulating and balancing diets for ruminants, maximizing nutrient synchronization, minimizing energy and nitrogen losses due to ruminal fermentation, and promoting greater efficiency of microbial synthesis [18].

We hypothesize that there are nutritional differences among the babaçu by-products, which will directly affect ruminal degradation. Thus, the aim of this study was to evaluate the chemical composition and *in situ* degradability of babassu by-products.

## 2. Materials and Methods

### 2.1. Laboratory Analyses

Laboratory analyses were conducted at the Laboratory of Animal Products (LAPOA) and the Laboratory of Bromatology and Animal Nutrition, both belonging to the Center for Agricultural and Environmental Sciences at the Universidade Federal do Maranhão, Chapadinha, Maranhão, Brazil (03°44′33″S latitude and 43°21′21″W longitude).

### 2.2. Samples and Experimental Design

The experiment was conducted in a completely randomized experimental design, with 4 by-products from babassu processing (cake, greasy, fine flour, and 95 *µ*m flour) (treatments) and 5 replications. The evaluated by-products were obtained from babassu processing by the company Florestas Brasileiras S.A., Itapecuru Mirim, Maranhão, Brazil.

**Figure 1:**
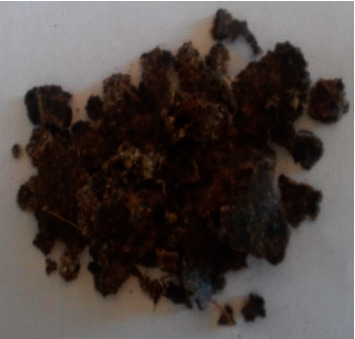
Babassu cake.

To obtain the babassu cake, the babassu almond is washed, weighed, and ground to facilitate cooking and pressing. Cooking releases the oil particles contained in the cells; in addition, it eliminates toxins that may be present in the almond. After cooking, the almond is pressed to extract the oil, leaving the babassu cake (Figure 1).

**Figure 2:**
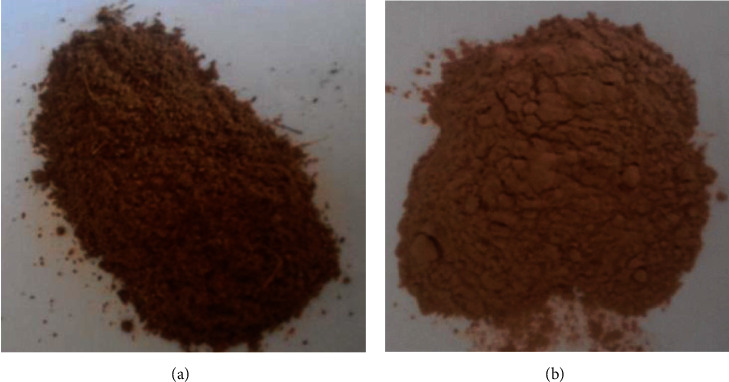
Fine babassu flour (a) and 95 *µ*m babassu flour (b).

Babassu flour was obtained through the process of classifying and grinding the mesocarp as part of the full use of the coconut, in which to obtain this, it is necessary to remove impurities from the mesocarp, which is done with the aid of sieves, which they have holes with different diameters, which give them different particle sizes for commercialization. After sieving, the mesocarp is moistened and roasted. After this process, flours are generated, which differ in texture and particle size (Figure 2). At a commercial level, the babassu flour used in these studies was classified according to the following granulometry: premium flour (less than 150 *μ*—premium type) and fine flour (150 *μ*—type I).

**Figure 3:**
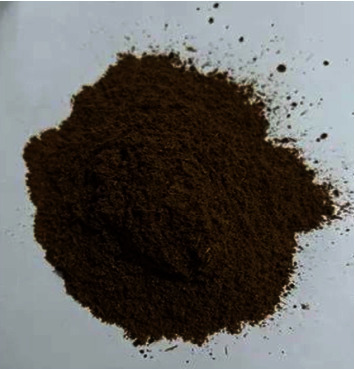
Greasy babassu by-product.

Greasy babassu by-product (Figure 3) (also known as borra de babaçu) is obtained after the first step of the refining process (degumming) that produces oil for human consumption.

### 2.3. Chemical Composition

Samples were predried for 72 h in a forced-air oven at 60 ± 5°C. Subsequently, the samples were ground (Wiley mill, Marconi, MA—580, Piracicaba, Brazil). Chemical composition was determined by analyzing dry matter (DM), ash, organic matter (OM), crude protein (CP), and ether extract (EE) following the methodology described by AOAC [19]. Neutral detergent fiber (NDF) and acid detergent fiber (ADF) were determined according to Soest et al. [20], with modifications proposed by Senger et al. [21], using autoclaving at 110°C for 40 min. Lignin (LIG) was determined by treating the acid detergent fiber residue with 72% sulfuric acid [22]. Cellulose (CEL) and hemicellulose (HEM) fractions were estimated from the fibrous fractions.

The contents of total carbohydrates (TC; [23]), nonfiber carbohydrates (NFC; [24]), and total digestible nutrients (TDN; [25]) were also determined.

### 2.4. *In Situ* Degradability

Samples (4 g) were placed in bags (13 × 4 cm) made of nonwoven fabric with a weight of 100 g/m^2^ [26]. For *in situ* incubation, a rumen-fistulated bovine with an average live weight of 400 kg was used. The bags were inserted into the rumen simultaneously and removed at 0, 3, 6, 12, 24, and 72 hours. Immediately after removal from the rumen, the bags were immersed in cold water and then manually washed in running water at room temperature. The bags from time zero were used to determine the soluble fraction (fraction a); they were placed in a water bath at 39°C for one hour and then washed together with bags from other incubation times until the water became clear.

The bags were dried in an oven (60 ± 5°C; 72 hours) and weighed. The residues remaining in the bags were analyzed for dry matter, crude protein [19], and neutral detergent fiber [27] contents. The estimation of potential (PD) and effective (ED) *in situ* degradability parameters of dry matter and crude protein was determined based on the method proposed by Ørskov and McDonald [28]:

(1) $$\begin{array}{c} \text{PD}=a+b\left(1-\;\exp\;-\text{ct}\right),\\ \text{ED}=a+\frac{\left(b\times c\right)}{\left(c+k\right)}, \end{array}$$

where *a* = water-soluble fraction; *b* = water-insoluble fraction, but potentially degradable; *c* = degradation rate of fraction *b* in the nylon bag after time zero, *t* = incubation time (h), and *k* = passage rate (passage rates of 2, 5, and 8% per hour were considered [29]).

The degradability of NDF was estimated using the model proposed by Waldo et al. [30]:

(2) $$\begin{array}{c} \text{Rt}=b\times e-\text{ct}+I, \end{array}$$

where Rt = residue at time *t* (hours); *b* = fraction of potentially degradable NDF in the rumen; *c* = degradation rate of fraction *b*; I = indigestible fraction.

After adjusting the NDF degradation equation, fractions were standardized using the equations [30]:

(3) $$\begin{array}{c} \text{PF}=\frac{b}{\left(b+I\right)}\times 100,\\ \text{IF}=\frac{I}{\left(b+I\right)}\times 100, \end{array}$$

where PF = standardized potentially degradable fraction (%); IF = standardized indigestible fraction (%).

### 2.5. Statistical Analysis

The data were subjected to analysis of variance using PROC MEANS. The parameters a, *b*, and *c* and the *in situ* degradability curves of the nutritional principles were analyzed using the procedure for nonlinear models (PROC NLIN). Means were compared using the Tukey test, considering *α* = 0.05, using the PROC MIXED procedure. All analyses were performed using the statistical software SAS [31], version 9.0.

## 3. Results and Discussion

The differences in the composition of babassu by-products are primarily due to the type of processing used to obtain each by-product, combined with variations in climate and region of the samples used, as these variables can alter the chemical composition of this ingredient. The different babassu by-products showed differences in chemical composition (*P* < 0.05), except for hemicellulose (*P*=0.0677) (Table 1).

It was possible to observe that the greasy presented a higher content of DM (*P* < 0.05) (Table 1), which is related to its processing, obtained from residues of the epicarp (outer fibrous part; 12% of the total fruit weight) and mesocarp (grainy starch-rich layer located between the epicarp and the endocarp (23% of the total fruit weight)) [32], where these components have in their constitution a lignified, hard, impermeable, and resistant tissue, which would limit feed intake by small ruminants. To the best of our knowledge, there are no studies evaluating the inclusion of greasy in ruminant diets, making it necessary and pertinent for future studies to elucidate the current literary limitations regarding this ingredient.

The babassu cake showed higher contents of ash and CP (*P* < 0.05) compared to the other by-products (Table 1). The values obtained are below those reported by Castro et al. [33] with 205.8 g/kg CP and 44.9 g/kg ash when using babassu cake in sheep diets and by Castro et al. [34] with 288.6 g/kg CP and 51.3 g/kg ash when incorporating babassu cake into cattle diets. The above-mentioned authors observed an increase in the intake of these nutrients by animals with the increase in the levels of babassu cake in the diets offered.

The CP content of babassu cake is above the range of 6 to 8% recommended by Van Soest [25] for effective ruminal microbial fermentation, with good nitrogen availability, which can enhance fiber digestion and enable increased intake by ruminants. Babassu cake is the main by-product derived from babassu, being widely used for protein enrichment of silages [17, 35] and diets offered to ruminants as a source of roughage or as a substitute for soybean meal [33, 34, 36].

The fine flour showed higher contents of EE and LIG (*P* < 0.05) compared to the other by-products (Table 1), while the 95 *µ*m flour showed higher levels of TC and TDN (*P* < 0.05) in its composition compared to the other studied by-products. The results obtained differ from those found by Sá et al. [37] when evaluating the use of babassu endocarp flour in the formulation of diets for sheep. The authors found 86.6 g/kg of EE, 206.7 g/kg of lignin, 843.1 g/kg of TC, and 474 g/kg of TDN in the endocarp flour I evaluated. According to the authors, endocarp flour I has a fine and powdery particle size, as it is separated by a suction system, being composed only of the endocarp, whereas endocarp flour II has a coarse particle size rich in fibrous bundles and small almond pieces. Thus, we can infer, based on the characteristics of the flours studied, that the flours evaluated in this study can be called endocarp flour I (95 *µ*m flour) and endocarp flour II (fine flour).

The difference in EE content between fine flour and 95 *µ*m flour may be due to the type of processing used to obtain them, as they differ in texture and particle size, with 95 *µ*m flour classified as premium flour primarily intended for human consumption. The EE content of 95 *µ*m flour is below the maximum level (70 g/kg of EE) recommended by Van Soest [25] as limiting to ruminal fermentation. However, the EE levels observed for fine flour, greasy, and cake are above this threshold, deserving careful attention when formulating feed with these by-products.

Higher values of NDF and ADF were presented by babassu cake and fine flour compared to the other babassu by-products (*P* < 0.05) (Table 1). The results obtained are higher than those obtained by Dutra Santos et al. [38] for fine flour (644.0 g/kg of NDF and 390.2 g/kg of ADF) and by Castro et al. [34] for babassu cake (595.0 g/kg of NDF and 303.9 g/kg of ADF). The observed results for NDF and ADF contents may be due to contaminations during the extraction of mesocarp by epicarp and endocarp, which are fibrous [39]. The high content of fibrous components classifies babassu cake and fine flour as fibrous feedstuffs, whose characteristics may limit the inclusion of these ingredients in diets offered to ruminants, mainly limiting their energy content [37].

The observed NDF and ADF results are above the 60% NDF recommended by Van Soest [25] and the range presented by Almeida et al. [40] for ADF (20 to 30%) for use in ruminant diets. Thus, we can infer that the high proportions of NDF and ADF in babassu cake and fine flour indicate the presence of lignocellulosic constituents, which are less utilized by the animals and negatively correlated with dry matter intake and digestibility [41]. Babassu cake and fine flour also showed higher levels of cellulose (*P* < 0.05), which is correlated with the high levels of NDF and ADF present in these ingredients.

The greasy and 95 *µ*m flour show higher NFC content compared to the other babassu by-products (*P* < 0.05) (Table 1). This is likely related to the lower levels of fibrous constituents present in these by-products compared to babassu cake and fine flour. The NFC values obtained for the 95 *µ*m flour are higher than the 351.0 g/kg found by Santos et al. [14]. There are no studies presenting the chemical composition of greasy babaçu by-product.

The highest percentage of the water-soluble fraction (fraction a) of DM was presented by the 95 *µ*m flour (37.34%) compared to the other by-products (Table 2). This difference can be explained by the lower fiber content observed in the 95 *µ*m flour and its particle size, which provided greater solubility of the DM compared to the other by-products. Zeoula et al. [42] mention that the particle size of incubated feed may explain the high values of the soluble fraction due to losses in the washing process.

On the other hand, fine flour showed the highest average percentage (56.93%) for the water-insoluble but potentially degradable fraction (fraction b) (Table 2). This percentage is related to the higher levels of NDF, ADF, CEL, and lignin that this ingredient presented in its composition. The highest degradation rate of fraction b per hour (fraction c) observed in the greasy (8.84%/hour) (Table 2) possibly occurred due to the lower levels of NDF and ADF.

The 95 *µ*m flour and fine flour showed higher values of PD and ED of DM at the three passage rates (2%, 5%, and 8%/h) (Table 2). These results were a consequence of the characteristics of fractions “a” and “b” presented by the 95 *µ*m flour and fine flour and the high starch content they possess in their composition, as described by Cinelli et al. [43], where the authors observed 60.05% of starch for babassu flour, which according to Van Soest [25], being a nonstructural carbohydrate, can be extensively degraded by ruminal microorganisms.

It is observed that the ED for all by-products decreased as the passage rate increased, corroborating Costa et al. [44], who emphasize that this result is due to the shorter time the feed remains in the rumen, thus reducing the time ruminal microorganisms can act. The ED values of the 95 *µ*m flour and fine flour are above those described by Sousa et al. [39] in *in vitro* degradability tests, who observed values for ED of DM between 27.76 and 34.01% for type I mesocarp flour, and between 32.32 and 39.48%/hour for type II mesocarp flour. This difference may be attributed to the higher content of fibrous constituents found by the authors, compared to the results obtained in this study. On the other hand, the ED values of babassu cake, at different passage rates, are higher than those described by Farias et al. [45], 25.75 and 33.64%/hour.

Regarding the parameters of protein degradation (Table 2), the 95 *µ*m flour presents a higher value of fraction “*a*,” which may be related to its particle size, leading to greater losses during bag washing. Nocek [26] emphasized that particle losses during bag washing can lead to large variations (physical disappearance), which seems to have occurred with the 95 *µ*m flour.

For fractions “*b*” and “*c*” of protein, the greasy and the 95 *µ*m flour showed higher values compared to the fine flour and cake. The higher values of this fraction presented by the greasy and the 95 µm flour may result from the lower levels of fibrous constituents (Table 1) presented by these by-products and also from the smaller particle size of the 95 *µ*m flour. However, despite having the highest protein content (155.16 g/kg) among the analyzed by-products, the cake was the by-product that obtained the lowest value for fraction “*b*,” indicating that a large amount of protein in this by-product may be associated with NDF and ADF. Santos et al. [14], when including mesocarp flour of babassu in diets for sheep, observed that as the levels of babassu flour in the diets increased, the fiber content also increased, which led to a reduction in protein degradation, since fibrous components such as ADF and lignin are responsible for low digestion of the cell wall in the rumen.

The greasy and the 95 *µ*m flour showed much higher potential and effective degradability of crude protein compared to the fine flour and cake (Table 2). Although the groats and the 95 *µ*m flour has low CP levels (Table 1), the higher PD values observed for these by-products were due to the larger fractions “*b*” and “*c*,” and because of the lower levels of NDF, ADF, LIG, and higher NFC (Table 1) compared to the other by-products. Regarding ED, these results were mainly due to the values obtained in fractions “*a*,” “*b*,” and “*c*,” which are used in the equation to calculate ED; thus, the results follow the effects observed for these fractions.

The colonization time of the by-products was similar, averaging 5.77 hours (Table 3). This parameter is favored by the presence of readily fermentable substrates and by the physical and chemical characteristics of the sample's cell wall, capable of facilitating microbial colonization. The times obtained are lower than those obtained by Freitas et al. [46], who evaluated the flour and cake of babassu, obtaining times of 9.88 and 8.14 hours, respectively, for the flour and cake of babassu, above the values found in this study. The difference between the times may be related to the concentrations of NFC, which, in the study by Freitas et al. [46], are higher than those found in this study.

The PF and IF fractions originate from the degradability of NDF [17]. Thus, the highest percentages of the PF fraction were observed for the 95 *µ*m flour and fine flour, consequently presenting lower IF fractions (Table 3). The higher percentage of the PF fraction exhibited by the 95 *µ*m flour was due to its lower fiber content (Table 1), while the fine flour, although it has high fiber content, also showed a high percentage of the PF fraction, possibly due to the high starch content it contains. However, further research is needed to assess the starch content of the babassu by-products studied here.

The highest passage rate percentage (k) of NDF was observed for the 95 *µ*m flour (5.84%/hour). It is worth noting that passage rates above 5% are recommended for high-quality forages and diets formulated with a combination of forage and concentrate components [28]. The higher passage rate exhibited by the 95 *µ*m flour was likely facilitated by its lower fiber content (Table 1), allowing for faster ruminal degradation. On the other hand, the greasy showed a lower passage rate (1.93%), a value close to the 2% passage rate typical of low-quality feeds [28]. The lower passage rate of the greasy suggests that this by-product is less degradable and requires more time in the rumen to be utilized. Portela et al. [35] emphasized that the less degradable the fiber, the longer the feed remains in the rumen, consequently limiting feed intake due to the sensation of fullness.

There was no treatment × incubation time interaction for the degradation of DM, CP, and NDF (Table 4). Isolated effect was obtained for the evaluated by-products and incubation times (*P* < 0.0001) in the degradation of DM. The higher value of DM degradation presented by the 95 *µ*m flour is related to its lower fiber content and higher percentages of potential and effective degradation that this by-product presents, suggesting that this by-product undergoes greater degradation compared to others, regardless of the incubation time. It can be inferred that it is better utilized by rumen microbiota, as reported by Santos et al. [14] when including babassu mesocarp flour in sheep diets.

The by-products differed in terms of treatment in PB degradation (*P*=0.0235) (Table 4). The 95 *µ*m flour showed higher degradation compared to greasy, possibly due to particle size. The 95 *µ*m flour also exhibited greater NDF degradation compared to the other by-products (*P* < 0.0001) (Table 4), which are possibly related to the higher standardized potentially degradable fraction (PF) and higher passage rate (k). Additionally, the 95 *µ*m flour may have high starch content, which, as a nonstructural carbohydrate, is extensively degradable by rumen microorganisms [47].

The assessment of babassu by-products allows us to infer that, despite the nutritional composition and degradability exhibited by flour and cake, these by-products cannot completely replace traditional concentrates. However, they enable partial inclusion in animal diets, depending on the animals' requirements and ingredient prices.

## 4. Conclusions

Among the studied by-products, babassu cake presents superior chemical composition. However, the 95 *µ*m flour simultaneously exhibited satisfactory nutritional value and ruminal degradation, making it suitable for use as an additive or in partial substitution of other traditional concentrates, depending on availability and affordable prices.

## Figures and Tables

**Table 1:** Chemical composition of babassu by-products.

Variables (% DM)	Babassu by-products				SEM	*P* value
	Greasy	Cake	Fine flour	95 *µ*m flour		
Dry matter^*∗*^	91.60^a^	88.92^c^	87.49^d^	89.79^b^	1.25	<0.0001
Ash	1.87^c^	4.18^a^	3.08^b^	3.14^b^	0.41	<0.0001
Crude protein	2.42^d^	15.51^a^	5.24^b^	3.02^c^	1.39	<0.0001
Ether extract	19.42^b^	11.80^c^	24.26^a^	0.48^d^	2.49	<0.0001
Neutral detergent fiber	27.90^c^	63.56^a^	66.05^a^	43.47^b^	21.40	<0.0001
Acid detergent fiber	16.73^c^	53.72^a^	54.78^a^	25.21^b^	7.58	<0.0001
Hemicellulose	11.17	9.84	11.26	18.26	22.37	0.0677
Cellulose	10.64^b^	43.33^a^	37.98^a^	13.98^b^	13.43	<0.0001
Lignin	6.08^c^	10.38^bc^	16.80^a^	11.23^b^	10.68	<0.0001
Total carbohydrates	76.27^b^	68.49^c^	67.41^c^	93.34^a^	3.02	<0.0001
Nonfiber carbohydrates	48.36^a^	4.92^b^	1.36^b^	49.87^a^	20.83	<0.0001
Total digestible nutrients	59.73^c^	46.99^d^	63.44^b^	64.70^a^	2.97	<0.0001

^*∗*^in % natural matter; DM = dry matter; SEM = standard error of the mean. Means followed by the same letters in the row do not differ significantly from each other according to Tukey's test at a 5% probability level. ^a,b,c,d^Means followed by the same letters in the row do not differ significantly from each other according to Tukey's test at a 5% probability level.

**Table 2:** *In situ* degradability of dry matter and crude protein of babassu by-products.

Variables	Babassu by-products				SEM
	Greasy	Cake	Fine flour	95 *µ*m flour	
*In situ degradability of dry matter*					
*a* (%)	25.63	21.60	20.63	37.34	3.84
*b* (%)	12.79	37.31	56.93	47.26	9.48
*c* (%/hora)	8.84	3.62	1.47	3.87	1.56
PD (%)	38.39	55.99	77.56	82.67	10.21
ED 2 (%/hour)	36.06	45.63	70.74	68.50	8.54
5 (%/hour)	33.80	37.27	63.11	57.96	7.33
8 (%/hour)	32.34	33.22	57.50	52.75	6.52
*R* ^2^	87.03	75.34	90.03	94.21	

*In situ degradability of crude protein*					
*a* (%)	18.92	24.33	21.23	28.20	2.01
*b* (%)	73.12	15.07	35.23	61.80	13.09
*c* (%/hora)	8.94	3.95	3.63	9.60	1.59
PD (%)	91.95	37.61	55.10	89.93	13.36
ED 2 (%/hour)	78.67	34.33	43.94	79.34	11.68
5 (%/hour)	65.81	30.98	36.05	68.84	9.83
8 (%/hour)	57.51	29.31	32.23	61.91	8.42
*R* ^2^	93.93	91.11	90.71	90.71	

*a* = water-soluble fraction (%); *b* = water-insoluble but potentially degradable fraction (%); *c* = degradation rate of fraction b (%/h); PD = potential degradation at 72 hours; ED = effective degradation; *R*^2^ = coefficient of determination; SEM = standard error of the mean.

**Table 3:** Colonization time (lag time), standardized potentially degradable fraction (PF), standardized undegradable fraction (IF), and passage rate (k) for neutral detergent fiber of babassu by-products.

Variables	Babassu by-products				SEM
	Greasy	Cake	Fine flour	95 *µ*m flour	
Lag time (hour)	5.64	5.87	5.81	5.77	0.05
PF (%)	51.58	45.86	66.49	69.90	5.78
IF (%)	48.42	54.14	33.51	30.10	5.78
k (%/hour)	1.93	4.15	3.35	5.84	0.82
*R* ^2^	97.40	99.03	95.20	90.77	

*R* ^2^ = coefficient of determination; SEM = standard error of the mean.

**Table 4:** Degradation (%) of dry matter (DM), crude protein (CP), and neutral detergent fiber (NDF) of babassu by-products.

Variables	By-products				SEM	Incubation times					SEM
	Greasy	Cake	Fine flour	95 *µ*m flour		3	6	12	24	72	
DM	37.33^b^	36.42^b^	36.21^b^	67.23^a^	2.42	36.62^B^	38.37^B^	41.59^B^	46.71^B^	58.20^A^	3.89
CP	26.86^b^	43.88^ab^	36.89^ab^	45.93^a^	2.43	31.90	36.95	35.96	36.19	50.99	3.27
NDF	33.43^b^	21.02^b^	28.41^b^	53.81^a^	2.63	31.02	27.40	31.67	37.03	44.47	2.97

*P* value											
	By-products	Incubation times	By-products × incubation times								

DM	<0.0001	<0.0001	0.1209								
CP	0.0235	0.1088	0.8141								
NDF	<0.0001	0.0785	0.2349								

By-products × incubation times = interaction effect between the by-products and the incubation times; SEM = standard error of the mean; a, b: means followed by different lowercase letters in the row differ the by-products; A, B: means followed by different uppercase letters in the row differ the incubation times; Significant at the 5% probability level by Tukey's test.

## Data Availability

The data that support the findings of this study are openly available in Universidade Federal do Maranhão at https://tede2.ufma.br/jspui/bitstream/tede/3277/2/YGOR-PORTELA.pdf.
